# Cervical myelopathy due to degenerative spondylolisthesis

**DOI:** 10.3109/03009734.2011.551932

**Published:** 2011-04-12

**Authors:** Tomoaki Koakutsu, Junko Nakajo, Naoki Morozumi, Takeshi Hoshikawa, Shinji Ogawa, Yushin Ishii

**Affiliations:** Department of Orthopaedic Surgery, Nishitaga National Hospital, Sendai, Japan

**Keywords:** Cervical spine, degenerative spondylolisthesis, myelopathy

## Abstract

**Objective:**

To investigate clinical-radiological features of cervical myelopathy due to degenerative spondylolisthesis (DSL).

**Methods:**

A total of 448 patients were operated for cervical myelopathy at Nishitaga National Hospital between 2000 and 2003. Of these patients, DSL at the symptomatic disc level was observed in 22 (4.9%) patients. Clinical features were investigated by medical records, and radiological features were investigated by radiographs.

**Results:**

Disc levels of DSL were C3/4 in 6 cases and C4/5 in 16 cases. Distance of anterior slippage was 2 to 5 mm (average 2.9 mm) in flexion position. Space available for the spinal cord (SAC) was 11 to 15 mm (average 12.8 mm) in flexion position and 11 to 18 mm (average14.6 mm) in extension position; 11 cases were reducible and 11 cases were irreducible in extension position. Myelograms demonstrated compression of spinal cord by the ligamentum flavum in extension position. Compression of spinal cord was not demonstrated in flexion position. C5-7 lordosis angle was lower than control. C5-7 range of motion (ROM) was reduced compared to controls. These alterations were statistically significant.

**Conclusions:**

DSL occurs in the mid-cervical spine. Lower cervical spine demonstrated restricted ROM and lower lordosis angle. Pathogenesis of cervical myelopathy due to DSL is compression of spinal cord by the ligamentum flavum in extension position and not by reduced SAC in flexion position.

## Introduction

Cervical myelopathy results from compression of the spinal cord by various degenerative processes of the spine. Kokubun et al. described the following seven spinal factors compressing the spinal cord: developmental stenosis, dynamic stenosis, disc herniation, segmental ossification of the posterior longitudinal ligament (OPLL), continuous OPLL, posterior spur, and calcification of ligamentum flavum. These factors were involved in 98% of cervical myelopathy cases (1). While it has long been suggested that degenerative spondylolisthesis (DSL) of the cervical spine may be a spinal factor (2), Kokubun et al. (1) did not include DSL as a spinal factor. The number of reports on cervical spondylotic myelopathy due to DSL has not been low (3–7), but no general consensus has been reached on the onset mechanism of spinal cord compression. The purpose of this study is to investigate clinical-radiological features of cervical myelopathy due to DSL.

## Patients and methods

Over a 4-year period from 2000 to 2003, 448 patients were operated for cervical myelopathy (excluding patients with rheumatoid arthritis, destructive spondylitis, and a past history of spinal trauma). Their medical charts and plain lateral functional X-rays of the cervical spine were reviewed, and DSL at the neurologically responsible disc level was observed in 22 patients (4.9%) (DSL group). There were 13 men and 9 women, with a mean ± SD age at the time of surgery of 74.8 ± 7.3 years (range 58–88 years). Of the patients who underwent surgery for cervical myelopathy during the same period, the same number of patients with dynamic stenosis (posterior slippage) or posterior spur as a spinal factor at the neurologically responsible disc level was randomly extracted as control (control group). For the control group, there were 14 men and 8 women with a mean ± SD age at the time of surgery of 67.3 ± 9.8 years (range 51–80 years). The mean age was significantly higher in the DSL group than in the control group (*p* = 0.0072, *t*-test).

Using plain lateral X-rays of the cervical spine which were taken with a tube-film distance of 1.8 m preoperatively, the following parameters were measured:

1. Disc level of DSL.
2. Distance of anterior slippage: the vertical distance between the posterior inferior angle of the slipped vertebra and the tangential line of the posterior surface of the caudal adjacent vertebra.
3. Space available for the spinal cord (SAC): the distance between the ventrolateral border of the vertebral arch of the slipped vertebra and the posterior superior angle of the lower adjacent vertebra.
4. C5-7 range of motion (ROM): according to Sasaki's method (8), the difference in the intersection angle of the tangential line of the posterior margin of vertebrae between extension and flexion positions.
5. C5-7 lordosis angle: according to Sumi's method (7), on plain lateral X-rays, the angle formed by the tangential line of the superior margin of C5 and the tangential line of the inferior margin of C7 in the neutral position was measured; lordosis was defined as ≥0° and kyphosis as <0°.

When accurate measurements were difficult because C7 overlapped the shoulder, C6 was used instead. Parameters 4) and 5) were also measured for the control group and subjected to statistical analysis (*t*-test, hazard ratio of 1%). For the DSL group, myelography and CT after myelography (CTM) in extension and flexion positions were used to investigate the pathogenesis of spinal cord compression.

## Results

### Disc level of DSL

Disc level of DSL was C3/4 in 6 patients and C4/5 in 16 patients; anterior slippage was in the mid-cervical spine in all patients.

### Distance of anterior slippage

The average distance of anterior slippage was 2.9 ± 0.8 mm (range 2–5 mm) in the flexion position, 1.1 ± 1.1 mm (range 0–3 mm) in the neutral position, and 0.6 ± 0.7 mm (range 0–2 mm) in the extension position. In 11 patients (50%), anterior slippage was reduced in the extension position. Seven patients (32%) also had developmental stenosis (anteroposterior diameter of the spinal canal ≤12 mm).

### Space available for the spinal cord (SAC)

The average SAC was 12.8 ± 1.2 mm (range 11–15 mm) in the flexion position and 14.6 ± 2.4 mm (range 11–18 mm) in the extension position. In the flexion position where distance of anterior slippage distance was the greatest, SAC was minimum.

### C5-7 range of motion (ROM)

The average C5-7 ROM was 7.3 ± 5.5° in the DSL group and 13.4 ± 7.0° in the control group. The ROM was significantly smaller in the DSL group (*p* = 0.0028).

### C5-7 lordosis angle

The average C5-7 lordosis angle was −0.4 ± 8.6° in the DSL group and 6.8 ± 5.9° in the control group. The C5-7 lordosis angle was significantly smaller in the DSL group (*p* = 0.0030).

### Pathogenesis of spinal cord compression

**Figure 1.**
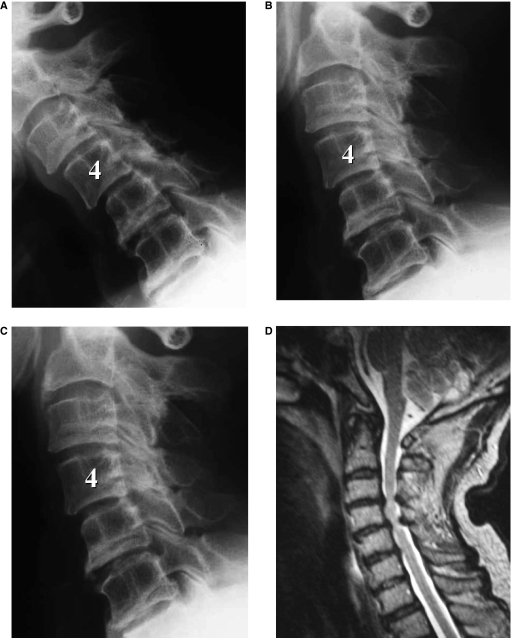

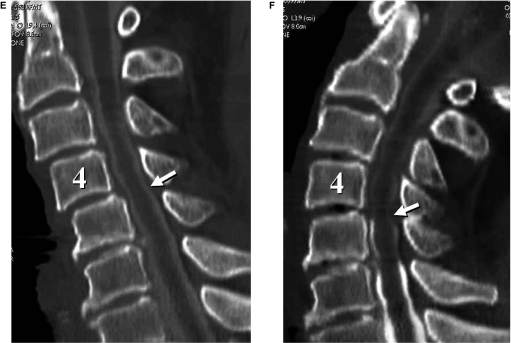
An 84-year-old man with C4/5 myelopathy. A, B, and C: Plain X-ray (A: flexion position, B: neutral position, and C: extension position). In the flexion position, 2-mm anterior slippage in C3 and 3-mm anterior slippage in C4 are demonstrated (A). In the extension position, C3 anterior slippage is reduced, and C4 anterior slippage is 1 mm (C). Space available for the spinal cord at C4/5 in the flexion and extension positions is 12 and 15 mm, respectively. C5-7 range of motion and lordosis angles are 3° and 4°, respectively. D: MRI showed spinal cord compression at C3/4 and C4/5. E and F: CT after myelography (E: flexion position and F: extension position). In the flexion position with maximum anterior slippage and osseous stenosis, deformation due to spinal cord compression is not confirmed (E). In the extension position with minimum anterior slippage and osseous stenosis, findings indicative of spinal cord compression from the posterior direction due to the buckling of the ligamentum flavum are confirmed (F).

A typical DSL patient is presented in Figure 1. In all patients in the DSL group, myelography and CTM did not show spinal cord compression in the flexion position where SAC was minimum, but in the extension position where SAC was maximum, spinal cord compression caused by the protruded disc and the ligamentum flavum was confirmed.

## Discussion

From antiquity, DSL has been known to cause spinal cord compression (2). Sasaki (8) examined X-rays of the cervical spine of 500 healthy individuals in different age-groups and reported that age-related degeneration occurred most commonly in C5/6 and that C4/5 ROM increased with age in a compensatory manner. Hayashi et al. (5) also documented that age-related degeneration lowered the ROM of C5/6 and C6/7 but increased the ROM of C3/4 and C4/5 in a compensatory manner, resulting in vertebral slippage. In particular, they found that posterior slippage occurred in extension position, but they did not mention anterior slippage. Sumi et al. (7) grouped cervical myelopathy patients into three groups based on C5-7 alignment (lordosis group, straight group, and kyphosis group) and confirmed C4 DSL in 41.2% of the straight group and 61.5% of the kyphosis group. Suga et al. (6) reported that C4 DSL occurred in the elderly due to decreased C5-7 ROM and forward-leaning body posture, and they emphasized the importance of C4 DSL in elderly patients with cervical myelopathy. In this study, the C5-7 ROM and the C5-7 lordosis angle in the patients with DSL were significantly lower than in the patients with other spinal factors, and DSL occurred at C3 and C4. The above findings suggest that DSL occurs in mid-cervical spine due to decreased lordosis angle and ROM of the lower cervical spine.

As for the clinical state of spinal cord compression due to DSL, Penning (3) and Epstein et al. (4) stated that spinal cord compression was caused by osseous spinal canal stenosis of the vertebral arch of slipped vertebrae and the posterior superior angle of lower cervical vertebrae in the flexion position where slippage was the greatest. In this study, myelography and CTM did not show findings indicative of spinal cord compression in the flexion position where anterior slippage and osseous spinal canal stenosis were the greatest, but in the extension position, spinal cord compression by the invagination of the ligamentum flavum into the canal was seen without osseous spinal canal stenosis. Spinal cord compression involving DSL was considered to be due to the buckling of the ligamentum flavum in the extension position irrespective of osseous stenosis. MRI for suspected cervical myelopathy due to DSL should be performed in extension position because spinal cord compression may not be observed when the cervical spine is in the flexion position. In the present series, long-term surgical results have not been observed, but when taking into account the onset mechanism of spinal cord compression, posterior decompression (either laminectomy or laminoplasty) is rational.

## References

[1] Kokubun S, Sato T, Ishii Y, Tanaka Y (1996). Cervical myelopathy in the Japanese. Clin Orthop..

[2] Clarke E, Little JH (1955). Cervical myelopathy; a contribution to its pathogenesis. Neurology..

[3] Penning L (1962). Some aspects of plain radiography of the cervical spine in chronic myelopathy. Neurology..

[4] Epstein JA, Carras R, Epstein BS, Levine LS (1970). Myelopathy in cervical spondylosis with vertebral subluxation and hyperlordosis. J Neurosurg..

[5] Hayashi H, Okada K, Hamada M, Tada K, Ueno R (1987). Etiologic factors of myelopathy. A radiologic evaluation of the aging changes in the cervical spine. Clin Orthop..

[6] Suga T, Miyazaki M, Akiyama K, Yoshida S, Mihara S (2005). Cervical spondylotic myelopathy in aged patients. Importance of C4 anterolisthesis. Rinshoseikeigeka..

[7] Sumi M, Kataoka O, Sawamura S, Ikeda M, Mukai H (1998). Radiological analysis of cervical spondylotic myelopathy. Rinshoseikeigeka.

[8] Sasaki A (1980). Radiology of normal cervical spine. J Jpn Orthop Assoc.

